# Factors influencing the work passion of Chinese community health service workers: an investigation in five provinces

**DOI:** 10.1186/1471-2296-15-77

**Published:** 2014-05-01

**Authors:** Zhenni Luo, Xue Bai, Rui Min, Changmin Tang, Pengqian Fang

**Affiliations:** 1 School of Health and Medicine Management, Tongji Medical College, Huazhong University of Science and Technology, 13 Hangkong Road, Wuhan, Qiaokou District 430030, China; 2 School of Health Management, Guangzhou Medical University, Guangzhou 510182, China; 3 School of Management, Hubei University of Chinese Medicine, Wuhan 430065, China

**Keywords:** Community health services, Community health workers, Work passion, Health care reform, China

## Abstract

**Background:**

After the implementation of new healthcare reform, Chinese government paid increasing attention to developing community health service (CHS). The current focus is mainly on cultivating community general practitioners but paying less attention to the working status and occupational demands of in-service CHS workers. Work passion is playing an important role for medical workers. With work passion, CHS workers’ team will become more stable and more effective, ensuring the sustainable development of CHS system. At present, the work passion of CHS workers is relatively low. Studying on influencing factors of work passion of CHS workers, promoting their work passion, and making them keep enthusiasm for work are significant.

**Methods:**

A total of 100 CHS organizations were sampled randomly in 10 cities from 5 Chinese provinces for this study. A total of 3450 CHS workers from these CHS institutions took part in the surveys. Questionnaires were used to collect data, including socio-demographic information, work passion and opinion on influencing causes, and work-related satisfaction. Pearson chi-square statistical method was used to identify the factors related to CHS workers’ work passion. Binary logistic regression was performed to determine the significant factors that influence CHS workers’ work passion.

**Results:**

A total of 38.77% of those who accomplished the questionnaire expressed that they didn’t have passion for current work. The related factors that influence CHS workers’ work passion are (1) socio-demographic factors such as age, and years of employment, and (2) other work-related factors such as learning and training opportunities, compensation packages, work stress, and personal development opportunities. CHS workers were most dissatisfied with the balance between remuneration and workload, job promotion opportunities.

**Conclusions:**

Based on the results, the government should concern for CHS workers’ working status and work-related demands, pay more attention and meet their demands for reasonable compensation packages and self-development, balance the income and workload, provide more learning and training opportunities and personal development opportunities for CHS workers, in order to promote CHS workers’ work satisfaction, improve their work passion and enthusiasm.

## Background

Healthcare system has three levels in urban area in China. The top level includes large-scale tertiary hospitals; the middle level includes medium-scale secondary hospitals; and the basic and primary level includes community health services (CHS) institutions providing primary healthcare services. CHS institutions are small-scale medical institutions located in urban communities in China, usually providing out-patient diagnosis and treatment, and in-patient care and treatment of common disease and chornic disease for community resident. For China and many other countries in the world, CHS system is an important part of healthcare system.

Many countries have developed an efficient CHS system, as evidenced by the involvement of stable and professional CHS workers and the provision of good healthcare services to community residents [1]. The CHS, which is mainly concerned with general practice and community care [2], can help to optimize the allocation of health resources and offer convenient and humanistic medical services while helping control the high costs of medical treatment [3,4]. Many countries provide satisfactory primary medical services to residents by producing well-trained community general practitioners and community nurses [5-7]. However, in China, the education and development mechanism of community general practitioners and nurses is not good enough [8].

Differences in local conditions create different structures and patterns of CHS system. Many countries have been prioritizing the developing CHS, and China is no exception. In 2006, the state council promulgated “Guidelines on the Development of City CHS [No. 10 (2006) of the State Council] and “Guidelines on Strengthening the Role of Urban Community Health Personnel” [No. 69 (2006) of the Ministry of Human Resources and Social Security]. The goals were to develop an improved urban CHS system for China, strengthen the role of community health human resources, and improve general practice and community care. In 2009, China began to implement another set of medical and health system reform that focused on developing CHS system as the foundation and center of health work in the future, presented applicable measures, and set the goal of cultivating, developing, and stabilizing CHS talent [8].

At present, urban CHS of China has attained much progress and development. As of April 2013, a total of 33,990 CHS institutions have been organized throughout the country [9]. In the first quarter of 2013, CHS institutions have diagnosed a total of 194,470,000 people and achieved a hospital bed utilization rate of 58.8%, with an average length of stay of 9.7 days [10]. However, the development of CHS human resources is limited, as well as the technical capability. The income and training opportunities of CHS workers are relatively less than those of medical workers in hospitals.

Chinese CHS provides basic diagnostic and treatment services for disease prevention, keeping fit, recuperating health, health education, family planning technical service, common diseases, frequently encountered diseases, and chronic diseases [11]. However, according to the former studies on the residents, only less than 30% of the residents are willing to choose CHS as the preferred option for treatment. Many residents who had received services from CHS once are not very satisfied [12,13], because they do not have enough confidence in CHS [14] and think that the technical capability of CHS is insufficient [15,16].

According to the former studies, CHS workers have relatively low work passion, low work efficiency, low work satisfaction, high demission rate, are not well educated, and possess poor professional skills, among others [17,18]. However, CHS workers are crucial for the excellent performance of the CHS [19]. Their working situation is directly related to community medical care safety, service quality, worker-patient relationship, patient satisfaction, and management and operational aspects of CHS institutions [20].

For one’ s work, passion is “a psychological state with the characteristics of strong and positive encouraging emotion, internal driving force, and putting into meaningful work with whole attention [21]”, and is having freshness and initiatives in work, having enthusiasm for work and keeping in a good working condition. Work passion is playing an important role for medical workers. With work passion, CHS workers’ team will become more stable and more effective, ensuring the sustainable development of CHS system. While, if CHS workers are lack of work passion, they are more likely to develop several problems such as low work efficiency, more emotional exhaustion, more job burnout, and more turnover intention [22]. All these problems will influence the quality of patients’ treatment and care. Thus, probing into the current factors that influence the work passion of CHS workers, promoting their work passion, and making them keep enthusiasm for work are significant.

The purpose of this study is to analyze and determine the main related factors that influence work passion of CHS workers by investigating CHS workers from five Chinese provinces. We aim to avoid work passion loss, arouse work passion, motivation and enthusiasm for work, promote work efficiency, and reduce job burnout and turnover intention, to provide high-quality medical services to community residents and meet the basic health service demands of community residents.

## Methods

This study was approved by the Tongji Medical College Ethics Committee of Huazhong University of Science and Technology (IRB No: FWA00007304). The respondents were properly informed, and their voluntary participation was sought. The respondents were assured of the confidentiality of their data and responses.

### Study population

A multi-stage sampling method was used to select the participating CHS institutions. In order to make the samples more representative, considering that China is a large area, we initially selected five provinces in eastern region, southern region, western region, northern region and central region of China respectively according to geographical locations. Subsequently, two cities were selected from each province. In order to reduce influence of different economy, these ten cities are at the similar economic development level. Finally, a list of all the CHS institutions was offered by health department in each city and 10 CHS institutions were randomly selected from each of the ten cities. Thus, this study comprised 100 CHS institutions.

In each selected CHS institution, all CHS workers were selected as respondents, including doctors, nurses, public health workers, and medical technicians. The total number of respondents was 3450, which comprised 801 from Hubei province, 633 from Guizhou province, 474 from Hebei province, 912 from Guangdong province, and 630 from Zhejiang province. A total of 3450 CHS workers were investigated and 3220 valid answer sheets were returned, resulting in a response rate of 93.33%. In order to ensure and control the quality of the questionnaires, one-on-one, face to face questionnaire survey method is taken.

### Questionnaire

The questionnaire comprised three parts, namely, socio-demographic information, work passion and influencing causes, and degree of work satisfaction. The items of the questionnaire were validated and the value of Cronbach’s Alpha is 0.875 for reliability statistics.

The socio-demographic information included gender, age, educational background, position, professional title, and years of employment.

The investigation on work passion and influencing causes included two parts. The first part is one question on whether CHS workers feel themselves having work passion for current work or not. The second part included questions on their opinions about the factors that may influence work passion, such as compensation packages, learning and training opportunities, personal development opportunities, interpersonal relationships and team atmosphere, work stress, workload, working conditions and environment, and opportunity to work autonomously. In each question of this part, two options were provided to express the opinion: 1, yes; 2, no.

Adapting the Minnesota Satisfaction Questionnaire and based on the actual situation of CHS workers in China, we developed a 10-item general work satisfaction inventory. The investigation on the degree of work satisfaction of CHS workers included satisfaction on the opportunities to develop capacity for work, decision-making ability of superior, the stability level of job, superior’s attitude toward subordinate, self-decision in completing the job, methods and measures for implementing organization policies, sense of achievement from work, working conditions, job promotion opportunities, and balance between remuneration and workload. A five-point Likert scale was used to rate satisfaction degree in these aspects, with “option1” denoting “very dissatisfied” and scoring 1 point; “ option 2” denoting “dissatisfied” and scoring 2 points; “option 3” denoting “moderate (not too bad)” and scoring 3 points; “ option 4” denoting “satisfied” and scoring 4 points; and “option 5” denoting “very satisfied” and scoring 5 points. Then, the score of work-related satisfaction degree was calculated.

### Statistical analysis

EpiData 3.1 was used to establish a database, and double machine inputting method was used to enter the collected data into the computer. PASW18.0 (SPSS) was used to perform related statistical data analysis. The socio-demographic factors of the investigated CHS workers were summarized using a descriptive statistical analysis method. Pearson chi-square statistical method was used to assess whether these socio-demographic factors and other factors were related to CHS workers’ work passion. Binary logistic regression was then performed to determine the significantly factors that influence work passion loss. The dependent variable included the following criteria: lose work passion (1) or have work passion (0) for current work. We substituted the variables with statistical significance determined from Pearson chi-square statistical test into the binary logistic regression model for calculation (*P* < 0.05). Odds ratio (OR) was reported at a 95% confidence interval (CI) where appropriate. All of the tests were conducted at 5% significance level.

## Results

### Socio-demographic characteristics of CHS workers

The largest proportion of age of the investigated CHS workers ranged from 25 and 34 (37.0%), and about half of the investigated CHS workers were female (51.1%). The largest proportion of educational degree was junior college (56.2%), and the largest proportion of professional title was junior level title (67.7%; Table 1). In this study, different professional titles showed various professional skill levels of the medical workers. In China, a medical worker needs to undergo and pass a special qualification examination and assessment to achieve his (or her) professional title corresponding to a particular professional skill level.

**Table 1:** Socio-demographic features of the investigated CHS workers in five Chinese provinces

**Characteristic**	**CHS workers (n = 3220)**	**Percentage (%)**
Gender		
Male	1574	48.9
Female	1646	51.1
Age		
24 or below	728	22.6
25–34	1191	37.0
35–44	911	28.3
45–54	338	10.5
55 and above	52	1.6
Educational background		
Secondary technical school and below	808	25.1
Junior college	1810	56.2
Bachelor’s degree	596	18.5
Master’s degree or above	6	0.2
Position		
Doctor	1117	34.7
Nurse	1036	32.2
Public health worker	664	20.6
Medical technician	403	12.5
Professional title		
No title	206	6.4
Junior level title	2180	67.7
Middle level title	805	25.0
Senior level title	29	0.9
Years of employment		
1 year and below	374	11.6
2–5 years	776	24.1
6–10 years	586	18.2
11–15 years	473	14.7
16–20 years	438	13.6
20 years and above	573	17.8
Province		
Hubei	747	23.2
Guizhou	592	18.4
Hebei	441	13.7
Guangdong	850	26.4
Zhejiang	590	18.3

### Work passion of CHS workers

Results from the investigation conducted in five Chinese provinces showed that only 63.23% of the CHS workers expressed that they had work passion for current work but 36.77% expressed that they didn’t have work passion for current work. More than one-third of CHS workers lost their work passion and working enthusiasm.

### Assessment of socio-demographic factors influencing the work passion of CHS workers

Pearson chi-square test method was used to assess the correlation between all indexes of socio-demographic factors and work passion. The results showed that among the socio-demographic factors of CHS workers, age, professional title, and years of employment were significantly related to CHS workers’ work passion (*p* < 0.05; Table 2).

**Table 2:** Analysis of socio-demographic factors related to CHS workers’ work passion

**CHS workers**	**Don’t have work passion (n = 1184)**		**Have work passion (n = 2036)**		***χ***^**2**^	**P**
	**n**	**%**	**n**	**%**		
Gender						
Male	580	49.0	994	48.8		
Female	604	51.0	1042	51.2	0.008	0.973
Age						
24 or below	266	22.5	462	22.7		
25–34	397	33.5	794	39.0		
35–44	384	32.5	527	25.9		
45–54	126	10.6	212	10.4		
55 and above	11	0.9	41	2.0	22.906	**<0.001**
Educational background						
Secondary technical school and below	310	26.2	498	24.5		
Junior college	675	57.0	1135	55.7		
Bachelor’s degree	195	16.5	401	19.7		
Master’s degree or above	4	0.3	2	0.1	7.614	0.055
Position						
Doctor	432	36.5	685	33.6		
Nurse	384	32.4	652	32.0		
Public health worker	241	20.4	423	20.8		
Medical technician	127	10.7	276	13.6	6.636	0.084
Professional title						
No title	84	7.1	122	6.0		
Junior title	759	64.1	1421	69.8		
Middle title	329	27.8	476	23.4		
Senior title	12	1.0	17	0.8	11.085	**0.011**
Years of employment						
1 year and below	146	12.3	228	11.2		
2–5 years	298	25.2	478	23.5		
6–10 years	192	16.2	394	19.4		
11–15 years	171	14.5	302	14.8		
16–20 years	185	15.6	253	12.4		
20 years and above	192	16.2	381	18.7	14.092	**0.015**
Province						
Hubei	283	23.9	464	22.8		
Guizhou	213	18.0	379	18.6		
Hebei	177	15.0	264	13.0		
Guangdong	306	25.8	544	26.7		
Zhejiang	205	17.3	385	18.9	3.964	0.411

### Assessment of work-related factors influencing the work passion of CHS workers

Pearson chi-square test method was also used to assess the correlation between work-related factors and work passion. The results showed that interpersonal relationships and team atmosphere, learning and training opportunities, compensation packages, opportunity to work autonomously, work stress, and personal development opportunities were significantly related to CHS workers’ work passion (P < 0.05; Table 3).

**Table 3:** Assessment and analysis of the work-related factors influencing work passion of CHS workers

**Assessment on factors may influence work passion (result in work passion loss)**	**Don’t have work passion (n = 1184)**		**Have work passion (n = 2036)**		**Total**		***χ***^**2**^	**p**
	**n**	**%**	**n**	**%**	**n**	**%**		
Interpersonal relationships and team atmosphere								
Yes	745	62.9	1006	49.4	1751	54.4		
No	439	37.1	1030	50.6	1469	45.6	55.093	**<0.001**
Working conditions and environment								
Yes	635	53.6	1022	50.2	1657	51.5		
No	549	46.4	1014	49.8	1563	48.5	3.537	0.060
Learning and training opportunities								
Yes	728	61.5	1021	50.1	1749	54.3		
No	456	38.5	1015	49.9	1471	45.7	38.792	**<0.001**
Compensation packages								
Yes	854	72.1	1254	61.6	2108	65.5		
No	330	27.9	782	38.4	1112	34.5	36.766	**<0.001**
Opportunity to work autonomously								
Yes	824	69.6	1330	65.3	2154	66.9		
No	360	30.4	706	34.7	1066	33.1	6.165	**0.013**
Workload								
Yes	741	62.6	1203	59.1	1944	60.4		
No	443	37.4	833	40.9	1276	39.6	3.829	0.05
Work stress								
Yes	657	55.5	890	43.7	1547	48.0		
No	527	44.5	1146	56.3	1673	52.0	41.596	**<0.001**
Personal development opportunities								
Yes	723	61.1	1002	49.2	1725	53.6		
No	461	38.9	1034	50.8	1495	46.4	42.266	**<0.001**

### Analysis of multiple factors influencing work passion of CHS workers

To further analyze and determine the factors influencing CHS workers’ work passion, we analyzed the significantly related factors (*p* < 0.05) described in the preceding analysis (Pearson chi-square test) by using binary logistic regression method. Dependent variables included the following: having no work passion (*Y* = 1) or having work passion (*Y* = 0) for current job. The results of multiple factors analysis showed that age and years of employment were significantly correlated to CHS workers’ work passion loss. The work passion loss probability of CHS workers aged between 25 and 34 was 1.921 times higher than that of CHS workers aged 24 and below (OR = 1.921, 95% CI = 1.192-3.095, *p* = 0.007). The work passion loss probability of CHS workers with 2 to 5 years and 6 to 10 years of employment was 1.532 times higher (OR = 1.532, 95% CI = 1.067-2.165, *p* = 0.011) and 2.043 times higher (OR = 2.043, 95% CI = 1.215-3.178, *p* = 0.006) than that of CHS workers with less than one year of employment, respectively. In addition, the results showed that learning and training opportunities, compensation packages, work stress, and personal development opportunities had positive correlations with the work passion of CHS workers. The work passion loss probability of CHS workers who considered learning and training opportunities as the influencing factor of work passion was 1.460 times higher than that of other CHS workers (OR = 1.460, 95% CI = 1.053 2.024, *p* = 0.023). The work passion loss probability of CHS workers who considered compensation packages as the influencing factor of work passion was 1.591 times higher than that of other CHS workers (OR = 1.591, 95% CI = 1.084-2.335, *p* = 0.018). The work passion loss probability of CHS workers who considered work stress as the influencing factor of work passion was 1.394 times higher than that of other CHS workers (OR = 1.394, 95% CI = 1.004-1.936, *p* = 0.047). The work passion loss probability of CHS workers who considered personal development opportunities as the influencing factor of work passion was 1.413 times higher than that of other CHS workers (OR = 1.413, 95% CI = 1.037-1.986, *p* = 0.033; Table 4).

**Table 4:** Multiple factors analysis of the influencing factors of CHS workers’ work passion

**CHS workers**	**Reference to classification**	**B**	**p**	**OR**	**95%CI**
Age	24 or below				
25–34		0.653	**0.007**	1.921	(1.192,3.095)
35–44		-0.556	0.060	0.573	(0.321,1.024)
45–54		-0.444	0.230	0.641	(0.310,1.325)
55 and above		0.167	0.810	1.182	(0.304,4.601)
Professional title	No title				
Junior title		-0.020	0.947	0.980	(0.544.1.767)
Middle title		-0.604	0.070	0.546	(0.284,1.051)
Senior title		-1.264	0.108	0.283	(0.061,1.318)
Years of employment	1 year and below				
2–5 years		-0.669	**0.011**	1.532	(1.067,2.165)
6–10 years		-0.888	**0.006**	2.043	(1.215,3.178)
11–15 years		-0.001	0.997	0.999	(0.507,1.965)
16–20 years		0.104	0.782	1.109	(0.532,2.314)
20 years and above		0.652	0.092	1.920	(0.899,4.104)
Interpersonal relationships and Team atmosphere	no				
Yes		0.233	0.118	1.263	(0.943,1.691)
Learning and training opportunities	no				
Yes		0.379	**0.023**	1.460	(1.053,2.024)
Compensation packages	no				
Yes		0.465	**0.018**	1.591	(1.084,2.335)
Opportunity to work autonomously	no				
Yes		-0.073	0.717	0.930	(0.627,1.380)
Work stress	no				
Yes		0.332	**0.047**	1.394	(1.004,1.936)
Personal development opportunities	no				
Yes		0.369	**0.033**	1.413	(1.037,1.986)

### Work satisfaction of CHS workers

According to the statistical results of the work-related satisfaction degree scores in various aspects, CHS workers were more satisfied with the opportunities to develop capacity for work (average score: 3.67 ± 0.881), decision-making ability of superior (average score: 3.59 ± 0.855), and the stability level of job (average score: 3.55 ± 0.821), and were less satisfied with the balance between remuneration and workload (average score: 2.91 ± 1.047), job promotion opportunities (average score: 3.09 ± 0.865), and working conditions (average score: 3.27 ± 0.961; Table 5).

**Table 5:** Satisfaction degree of CHS workers in all aspects

**Option**	**N**	**Mean**	**SD**
Opportunities to develop capacity for work	3220	3.67	0.881
Decision-making ability of superior	3220	3.59	0.855
The stability level of job	3220	3.55	0.821
Superior’s attitude toward subordinate	3220	3.51	0.936
Self-decision in completing the job	3220	3.43	0.939
Methods and measures for implementing organization policies	3220	3.38	0.853
Sense of achievement from work	3220	3.34	0.906
Working conditions	3220	3.27	0.961
Job promotion opportunities	3220	3.09	0.865
Balance between remuneration and workload	3220	2.91	1.047

## Discussion

Work passion is a psychological state with the characteristics of strong and positive encouraging emotion, internal driving force, and putting into meaningful work with whole attention,and is having freshness and initiatives in work, having enthusiasm for work and keeping in a good working condition. As the providers of community health services, only CHS workers have work passion for their work can CHS workers team keep sustainability and stability, provide high-quality and efficient medical service for community residents. However, the result of our research showed that 36.77% of investigated CHS workers expressed that they didn’t have work passion for current work. Therefore, we need to further analyze the influencing factors of work passion loss to improve CHS workers’ work passion and enthusiasm.

Before discussing the findings of this study, let us study the results of related studies in different countries and regions. In an analysis of socio-demographic factors that influence the work passion, some studies show that age, years of employment, and educational background are correlated with work passion of medical workers [23,24]. In terms of the other factors that influence the work passion, studies show that work stress, working environment [25], learning opportunities, personal relationships, personal accomplishment [26], and being trusted and respected [27] are related to work passion of medical workers. Some studies also show that work passion has a significant negative correlation with work dissatisfaction, turnover intention [28], burnout syndrome [29], and emotional exhaustion [30]. Work passion and work satisfaction are two different kinds of psychological state, and work passion is significantly and positively influenced by work satisfaction.

The conclusions drawn in this study are similar to those drawn from other countries and regions. According to the results from multivariate analyses, it was showed that among the socio-demographic factors, age and years of employment were significantly related to the work passion of investigated CHS workers. The work passion loss probability of CHS workers between 24 and 34 years old was relatively high, as well as that of CHS workers with 2–10 years of employment. The reason may be that the new generation of young CHS workers was a bit more demanding for their career and self-development. They are more likely to be disappointed by the reality and loss work passion after working in CHS institutions for a while. According to the results from multivariate analyses, in work-related aspects, learning and training opportunities, compensation packages, work stress, and personal development opportunities all significantly influenced work passion loss of investigated CHS workers.

In order to avoid losing work passion, motivate work passion and enthusiasm of CHS workers, promote their work efficiency, reduce their job burnout and turnover intention, make CHS workers provide high-quality medical services to community residents, the government officials and CHS institutions administrators should pay attention to these influencing factors and focus on the demands of CHS workers. What should be done most by government officials and CHS institutions administrators at present mainly include four aspects. 1) The government officials and CHS institutions administrators should more concern for the working status and psychological state of CHS workers, especially the new generation of young CHS workers, and help them solve problems. 2) The government officials and CHS institutions administrators should improve the system of compensation packages, promote income levels, and balance the relationship between income, technical value and workload of CHS workers. 3) The CHS institutions administrators should properly assign and arrange work, improve working environment and conditions for CHS workers, in order to reduce their work stress. 4) More learning and training opportunities and more personal development opportunities should be provided and created to CHS workers by the government and CHS institutions administrators, in order to help them to improve their professional level and meet their individual development requirements.

According to the results on work satisfaction in many aspects, investigated CHS workers were most dissatisfied with the balance between remuneration and workload, and job promotion opportunities. These findings indicated that CHS workers thought that their current remuneration does not match their workload and were therefore dissatisfied with their current remuneration. CHS workers were also not satisfied with the current promotion opportunities and thought that the learning and training opportunities and personal development space provided currently to them do not meet their requirements. Work passion is significantly and positively influenced by work satisfaction [31]. Therefore, the results of investigation on work satisfaction also reflected the influencing factors of work passion of CHS workers and proved that remuneration and job promotion opportunities would influence work passion, which were similar with the results from multivariate analyses of factors influencing the work passion. Improving work satisfaction of CHS workers can help them avoid losing work passion. Government officials and CHS institutions administrators should pay attention and take measures to improve and promote the work satisfaction of CHS workers, especially balancing remuneration and workload and providing job promotion opportunities for CHS workers.

## Conclusions

The results of this study showed that over one-third of the investigated CHS workers didn’t have work passion for current work. The main influencing factors for this finding were work stress, compensation packages, learning and training opportunities, and personal development opportunities of CHS workers, which didn’t meet their requirements and dissatisfactory. This finding showed that work passion also related with socio-demographic factors of CHS workers, such as age and years of employment. We suggest government officials and CHS institutions administrators concern for CHS workers’ working status and work-related demands, pay attention to and meet their demands for reasonable compensation packages and self-development, balance the income and workload, provide more learning and training opportunities and personal development opportunities for CHS workers, in order to promote CHS workers’ work satisfaction, improve their work passion and enthusiasm, reduce the possibilities of job burnout and turnover intention, and make them determined to provide high-quality and efficient medical services to community residents. Government departments and organizations must prioritize these steps and fully understand the significance of arousing and maintaining the work passion of the CHS workers’ team for CHS development.

## Competing interests

We declare that we have no competing interests.

## Authors’ contributions

ZL, XB, RM, PF participated in literature search and the design of the study. ZL, XB, RM, CT took part in survey and data analysis. ZL contributed to data interpretation and the writing of the article. All authors have read and approved the final version.

## Pre-publication history

The pre-publication history for this paper can be accessed here:

http://www.biomedcentral.com/1471-2296/15/77/prepub
